# Protocol for the psychotherapeutic group intervention for facilitating posttraumatic growth in nonmetastatic breast cancer patients

**DOI:** 10.1186/s12905-016-0302-x

**Published:** 2016-05-04

**Authors:** Catarina Ramos, Isabel Leal, Richard G. Tedeschi

**Affiliations:** WJCR-William James Center for Research, ISPA – University Institute, Rua Jardim do Tabaco, 34, 1149-041 Lisbon, Portugal; Department of Psychology, University of North Carolina, Charlotte, NC USA

**Keywords:** breast cancer, group intervention, posttraumatic growth

## Abstract

**Background:**

Breast cancer can be perceived as a traumatic event with disturbing effects on psychological domains such as depression, anxiety, and Posttraumatic Stress Disorder. In contrast, growing evidence has shown that posttraumatic growth can occur as a result of coping with breast cancer. Challenging the assumptive world, deliberate rumination, and emotional disclosure are recognized as strong predictors of posttraumatic growth. Group interventions may also increase social support, distress disclosure, and posttraumatic growth. The aim of this study is to evaluate how group-based interventions can facilitate posttraumatic growth and promote improved psychosocial adjustment to breast cancer. This article describes the study protocol and the applied research methods.

**Methods:**

To measure the impact of a group-based intervention on posttraumatic growth, a multi-center randomized control trial was developed for Portuguese breast cancer patients. 205 women with nonmetastatic breast cancer (stages 1 to 3) were recruited for the study and were randomly assigned either to the experimental group, which participated in an 8-session group intervention, or to the control group. Psychosocial variables, which consisted of posttraumatic growth, illness perception, stressfulness of the event, Posttraumatic Stress Disorder, core beliefs, rumination, social support, and distress disclosure were measured at three time points. The designated points in time for the assessments were baseline, 6 months post-intervention, and follow-up (12 months after baseline).

**Discussion:**

This study is the first trial to assess the efficacy of a group-based intervention designed to facilitate posttraumatic growth following a breast cancer diagnosis. If proven to be effective, group-based intervention could be recommended as a complementary program to be included in hospital health-care and clinical practice.

**Trial registration:**

The trial was registered on 28/10/2013 at the Current Controlled Trials (ISRCTN02221709).

## Background

Breast cancer diagnosis can induce several negative psychological symptoms such as distress, anxiety, and even cancer-related Posttraumatic Stress Disorder (PTSD) [1, 2]. There are cognitive and affective symptoms that persist during the course of the treatment(s), with different levels and intensity over time [3, 4]. Along with the distress caused, the positive outcomes of having cancer have received substantial attention over the last decade [5, 6]. Currently, it is strongly recognized that individual struggles with the overwhelming effects of major life crises can lead to the perception of positive changes [7, 8].

The construct of posttraumatic growth (PTG) was first proposed by Tedeschi and Calhoun [8] to define an individual’s experience of positive change arising from “the struggle with the new reality in the aftermath of trauma that is crucial in determining the extent to which posttraumatic growth occurs” ([9], p. 5). Several studies reported PTG in women with a diagnosis of breast cancer within five years following being diagnosed [10–14].

PTG involves the reappraisal of traumatic events through different cognitive perspectives, with the main objective of reconstructing the basic assumptions or core beliefs about one’s self, the world, and the future, which are frequently disrupted in the aftermath of a traumatic event [15–17].

The challenge to one’s core beliefs appears to be a major antecedent to PTG, since the stressfulness of the event shatters the assumptive world and leads the survivor to engage in a cognitive process to understand what happened [15, 16, 18]. Moreover, within the cognitive process, the rumination related to the event is a crucial factor in the pathway of growth, since it is a key component with an intermediary function between the shattering of the assumptive world and PTG [19]. Rumination is generally defined as repetitive thinking or cognitive processing about certain information, including cognitive mechanisms such as problem solving or making sense of the situation [20–22]. Intrusive rumination is characterized by negative, maladaptive, distressing, and unwanted thoughts that occurred repeatedly and uncontrollably [18, 21]. In contrast, deliberate rumination consists of thoughts that occur deliberately, through which the individual purposefully re-examines the event and its inherent implications. Thus, during deliberate rumination, the individual is involved in intentional attempts to understand and assign a meaning to the traumatic event, which, in turn, can lead to increased awareness about the positive repercussions of the experience [18, 21]. According to some authors, deliberate thinking is the type of rumination that is most associated with the development of PTG [15, 20, 22].

The experience of intense distress in the aftermath of a breast cancer diagnosis, may lead to the need to seek social support for distress disclosure [23, 24]. Furthermore, it is emotional disclosure concerning illness-related stress within social relationships, which influences the reconstruction of one’s assumptive world, cognitive reappraisal, and fosters deliberate rumination about one’s experience with breast cancer [15, 23, 25]. These conditions have been recognized as key factors for the development of PTG [25], specifically among women with breast cancer [26].

The efficacy of psychosocial support groups in regards to the psychological well-being of breast cancer patients is demonstrated by a number of studies [27, 28]. Supportive-Expressive Therapy (SET) [29], is shown to be effective in reducing anxiety, depression, mood disturbance, PTSD symptoms [30–32], and pain perception [31, 33], in addition to improving quality of life [34], and enhancing survival time [28]. Cognitive-Behavior Stress Management therapy (CBSM) is proven to reduce anxiety, emotional distress, and intrusive thinking [35, 36]. At the physical level, CBSM is demonstrated to be effective in improving physical functioning [37] and reducing patients’ cortisol levels [3, 38].

Beyond the psychological and physical benefits that are already recognized, group interventions may be another way to promote PTG. There have been some psychosocial interventions that have previously assessed growth as an outcome of group-based emotional support for women being treated for breast cancer [36, 39–41].

Moreover, a recent meta-analysis reported several new interventions that were developed with the aim to promote growth in aftermath of adverse events [42]. A recent study demonstrated the effectiveness of an intervention to promote PTG in women with breast cancer by showing that the participants of a group intervention reported more PTG over a 6-month period, in comparison with control group [43]. Other interventions proved to be effective in fostering PTG with war veterans [44], college students [45], as well as cancer patients and their families [46]. This can be interpreted as evidence that PTG may be enhanced by participation in group-based interventions. In fact, a group setting can provide a forum to discuss personal experiences, review cognitive schemas, and promote core belief reconstruction, which are the main predictors of PTG. Moreover, the group members who have already undergone positive changes during their experience with cancer can serve as role models, and may be credible sources for fostering PTG in others [47].

Aside from the evidence of positive changes resulting from group interventions, to the best of our knowledge there have not yet been any group interventions specifically designed to facilitate PTG in breast cancer patients. Based on previous model of PTG [9, 48], we designed a group-based intervention program to facilitate posttraumatic growth. If proven to be effective and efficient, this program could be used in a health-care context as a complementary form of psychological treatment for breast cancer patients.

### Objectives

In the study we present the detailed research protocol of a randomized controlled trial (RCT) to assess the efficacy of a group intervention in facilitating PTG in breast cancer patients. Additionally, we address the association of PTG with other variables related to psychosocial adjustment, such as the style of rumination, the challenge of core beliefs, social support, PTSD, distress disclosure, and illness perception. Our second aim is to assess how psychosocial variables moderate the effect of group intervention in PTG reports. Finally, the third objective of our study uses a longitudinal design to assess the potential differences in PTG at three different points in time.

## Methods/design

**Fig. 1:**
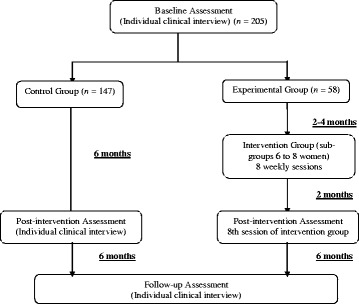
Experimental design and flow diagram of participants

The sample is composed of an experimental group and a control group of female Portuguese nonmetastic breast cancer patients recruited from multiple health-care centers. This study was conducted over the course of 8 sessions of group-based intervention. We used three points of measurement: baseline, 6 months (post group intervention), and 12 months after the baseline (follow-up) (Fig. 1). Both phases of the study utilized patient volunteers and were free of charge. The principal researcher led both the intervention groups and conducted the assessments at three designated measurement points.

The research was conducted in the oncology departments of three public hospitals (Centro da Mama - Centro Hospitalar de São João; Hospital de Santo António - Centro Hospitalar do Porto; Hospital de São Francisco Xavier - Centro Hospitalar de Lisboa Ocidental), a private clinic (Hospital da Luz), and an association for breast cancer patients (Movimento Vencer e Viver do Núcleo Regional do Sul da Liga Portuguesa Contra o Cancro). The public hospitals, private clinic, and breast cancer association are all located in Porto or Lisbon, the two largest cities in Portugal. Ethics approval was obtained from the Ethics Committee of each institution, namely, Comissão de Ética para a Saúde do Centro Hospitalar de São João; Comissão de Ética para a Saúde do Centro Hospitalar do Porto; Comissão de Ética do Centro Hospitalar de Lisboa Ocidental; Comissão de Ética para a Saúde do Hospital da Luz; Movimento Vencer e Viver do Núcleo Regional do Sul da Liga Portuguesa Contra o Cancro.

### Participants

For this study, we recruited 205 Portuguese women diagnosed with nonmetastatic breast cancer. Participants were also required to meet several additional inclusion criteria. The criteria included having their first and only breast cancer diagnosis between stages 1–3, having been diagnosed between January 2011 and the present; possessing no other type of cancer prior to or after the baseline point, being at least 18 years old, speaking and writing Portuguese fluently, and having no physical or mental disorder that could compromise participation in the study. Therefore, the exclusion criteria consisted of having another diagnosis of breast cancer or another type of cancer prior to or after the first assessment, psychological or physical comorbidities that make it unlikely that participants would complete the study (e.g. schizophrenia, major depression, personality disorder, etc.), and having substance abuse or other issues that may compromise patients’ participation in the study.

During recruitment, the contact information of the participants was collected through the consultation of their individual medical histories, in accordance with the data protection guidelines of the Portuguese Data Protection Authority (CNPD – Comissão Nacional de Protecção de Dados), which guarantee confidentiality through the appropriate methods of protecting patients’ data.

Alternatively, the multidisciplinary hospital team, including doctors, nurses, and psychologists, were notified about the study with the goal of informing participants.

Additionally, we selected participants who were scheduled to start treatment, who had already initiated any systematic treatment, or who had already finished any systematic treatment.

The research team leader contacted each participant in order to schedule the baseline assessment, which consisted of a semi-structured clinical interview.

### Power calculation

The sample size was calculated using G-Power 3.1. To calculate the power, the test for independent samples (significance level of 0.05) was used to compare the two groups. For a total sample of 70 subjects, this test showed a 95 % of power to detect a mean difference of 6.8 points between the control group and the group treated with psychotherapeutic intervention.

### Randomization method

Participants were selected by the researcher responsible for the research team and were subsequently assigned to one of two trial groups. The participants were randomly allocated either to the intervention group, where group intervention to facilitate PTG was performed, or to the control group, which was created in order to compare the effects of the intervention program. The main researcher stratified randomization, with participants being assigned to intervention groups of 6 to 8 participants each.

Moreover, the selection for the experimental group was made based solely upon the values obtained from the Post-Traumatic Stress Disorder Checklist – Civilian Version (PCL-C, 50), in the baseline assessment. In accordance with the theory of PTG [49], which asserts that PTG will occur only if the individual perceives an event as traumatic or stressful, the intervention group only included participants who reported medium or high PTSD values. Given the characteristics of the study and the nature of the intervention, it was not possible to conduct a blind study; however, the ethics committees consider that this is unlikely to influence the study’s outcomes.

### Control group

Participants in the control group underwent the standard treatment for breast cancer and did not receive any individual or group-based intervention on behalf of the study. However, they received the typical medical and psychological care provided to patients by the Department of Oncology at their respective hospitals (Treatment as Usual condition). Also, participants in the control group also attended three individual semi-structured clinical interviews, first for data collection at baseline, and subsequently at 6 and 12 months following the baseline interview.

### Intervention group

The participants who were allocated to the intervention group were divided into sub-groups of 6 to 8 women. They also completed individual interviews at the baseline and at the 12-month assessments. The 6-month assessment was carried out during the eighth session of the intervention group.

### Assessments

The first assessment of psychosocial and socio-demographic variables was conducted at the baseline for both groups and involved the completion of socio-demographic and psychosocial questionnaires, a brief explanation about the study’s objectives and procedures, as well as a brief semi-structured interview to assess each woman’s experience with breast cancer. During the interview, each participant signed the written informed consent, for participating in the study. The interview also aimed to evaluate any possible physical or mental conditions that might compromise the participation in the intervention or the control groups.

The assessment of psychosocial variables was repeated at two subsequent points in time. These were post-intervention (6 months after the baseline) and follow-up (12 months after the baseline). For both groups these assessments were conducted in a clinical interview setting, in a manner similar to that of the baseline assessment. However, for participants in the intervention group, the second assessment of the psychosocial variables was performed during the last session of the group intervention, with the exception of distress disclosure, which was assessed during the second group session (Table 1).

**Table 1:** Study measure Notes

	Questionnaires (measures)	Baseline	Intervention	Post-intervention 6 months^a^	Follow-up 12 months
Inclusion Criteria	Structured interview	X		X	X
	Demographic and medical data	X			
Primary Outcome	Posttraumatic growth (PTGI)	X		X	X
Secondary Outcomes	Posttraumatic Stress Disorder (PCL-C)	X		X	X
	Stressfulness of the event	X		X	X
	Illness perception (Brief IPQ)	X		X	X
	Challenge to core beliefs (CBI)	X		X	X
	Rumination (ERRI)			X	X
	Distress disclosure (DDI; Opener Scale)	X	X^b^	X	X
	Social support (ESSS)	X		X	X
Intervention Effectiveness	Questionnaire for assessment of quality of intervention and therapist		X		

Notes: PTGI – Posttraumatic Growth Inventory; PCL-C – Posttraumatic Stress Disorder Checklist – Civilian Version; Brief IPQ - Brief Illness Perception Questionnaire; CBI – Core Beliefs Inventory; ERRI – Event-Related Rumination Inventory; DDI – Distress Disclosure Inventory; ESSS – Social Support Satisfaction Scale.

^a^The second assessment is completed during the eighth session, to the intervention groups.

^b^The distress disclosure measures are applied in second session of the intervention program for the experimental group and in the second assessment, for the control group.

### Outcomes

1. PTG was the primary outcome evaluated and was measured through the Posttraumatic Growth Inventory (PTGI) [8]. This PTGI is a 21-item inventory that assesses the positive changes after a traumatic event, through a 6-point Likert scale ranging from 0 (*I did not experience this change as a result of my crisis*) to 5 (*I experienced this change to a very great degree as a result of my crisis*). It includes 5 domains of growth: personal strength, new possibilities, relating to others, appreciation of life, and spiritual change. The original scale [50] reported a satisfactory internal consistency for the total scale (α = .90) and for the sub-scales (Relating to Others, α = .85; New Possibilities, α = .84; Personal Strength, α = .72; Spiritual Change, α = .85; and Appreciation of Life, α = .67).The Portuguese translation of the PTGI for breast cancer patients [51] showed strong internal consistency for the total scale (Cronbach’s alpha = .937) and the sub-scales (Cronbach’s alpha ranged from .80 to .90).
2. The stressfulness of the event was assessed with 2 questions [19] on 7-point Likert scale ranging from 1 (*not at all stressful*) to 7 (*extremely stressful*). The questions were: “How stressful was the event for you at the time it happened?” and “How stressful is the event for you now?”.
3. The symptoms of PTSD were measured by PCL-C [52]. It contains 17 items arranged on a 5-point scale ranging from 1 (*not at all*) to 5 (*extremely*), which evaluates the distress and impact of breast cancer as a traumatic event through three subscales: intrusion, avoidance, and hyperarousal. This scale demonstrated strong internal consistency (Cronbach’s alpha = .97). In a sample of Portuguese women with breast cancer [53] the sub-scales also showed strong reliability (Cronbach’s alpha ranged from .86 to .91).
4. Illness Perception is primarily defined as the patient’s perceptions of breast cancer and was assessed using the Brief Illness Perception Questionnaire (Brief IPQ) [54], which is comprised of two parts. The first part consists of 8 items in analogical scale ranging from 0 to 10, and the second is an open question about the factors that contributed to one’s illness. The scale contains three dimensions, which are cognitive illness representations, emotional representations, and illness comprehensibility. Additionally, the scale is shown to have good psychometric properties [54].
5. The challenge to core beliefs was measured by the Core Beliefs Inventory (CBI) [15]. The CBI is a 9-item instrument that assesses the degree to which the traumatic event causes the re-evaluation of the assumptive world, including core beliefs about oneself, other people, the future, and the world, through a Likert scale ranging from 0 (*not at all*) and 5 (*to a very great degree*). This inventory showed good internal reliability (Cronbach’s alpha = .82) [15].
6. Rumination was assessed using the Event-Related Rumination Inventory (ERRI) [18]. The ERRI is comprised of two sub-scales, used to assess intrusive rumination and deliberate rumination. Each sub-scale consists of 20 items in a 4-point Likert scale ranging from 0 (*not at all*) to 3 (*often*) The participants completed the two scales during the baseline, 6 month, and 12 month assessments. At baseline the questions asked participants to reflect upon “the weeks immediately after the event”, and in the following assessments the instructions concerned “the last two weeks”. The two sub-scales had strong psychometric properties (intrusive rumination, α = .94; deliberate rumination, α = .88) [18].
7. Social support was evaluated by the Social Support Satisfaction Scale (ESSS) [55], which contains 15 statements describing four social support dimensions. The four dimensions are defined as relationship satisfaction, intimacy, satisfaction with family, and satisfaction with social activities. The assessment utilizes a Likert scale ranging from 0 (*strongly disagree*) to 5 (*strongly agree*). All of the sub-scales had satisfactory internal consistencies in the sample composed of Portuguese women with breast cancer (Cronbach’s alpha ranged from .56 to .85) [56].
8. Distress disclosure was evaluated with the Distress Disclosure Index (DDI) [57] and with 5 items from the Opener Scale [58, 59]. The DDI, measures the tendency of emotional expression and repression. Higher scores indicate a greater tendency for emotional expression, while lower scores indicate a greater likelihood for emotional repression. It consists of 12 items, which are assessed on 5-point Likert scale ranging from 1 (*strongly disagree*) to 5 (*strongly agree*). Cronbach’s alpha for the total score was .93. The Opener Scale consists of 5 items used to assess the extent to which the subject discussed each topic with her spouse or other intimate other during the previous week through a 5-point Likert scale varying from 1- (*Did not discuss at all*) to 5 (*Discussed fully and completely*). The Cronbach’s alpha for the Opener Scale was .83.

To assess the efficacy of the intervention, in the last session of the group intervention, we also used a questionnaire developed to assess one’s satisfaction with the intervention and with the therapist’s performance.

### Intervention

The group-based intervention took place between 2 months after the baseline assessment. The participants that were selected for the experimental group were randomly divided into subgroups of 6 to 8 participants. The group interventions lasted 8 weeks and occurred on a weekly basis, with a duration of 90 min per session. The intervention was designed in accordance with the model of PTG [9, 48], and the guidelines of several prior studies [44, 60, 61].

Each session included a breast cancer related topic, a theoretical exposition, and cognitive-behavioral psychological strategies, in order to accomplish certain objectives related to the psychosocial adjustment to breast cancer. Each session ended with a group discussion about the topic of the session.

It is noted that PTG is not mentioned during the intervention. This approach is supported by the model of PTG [49]. The detailed structure of each session is described below (Tables 2 and 3).

**Table 2:** Theme of each intervention session

Session	Theme
1	Psychoeducation and normalization of emotional reactions
2	Facilitating of emotional disclosure and communication
3	Practice emotional self-regulation skills
4	Fears and concerns related to breast cancer
5	Balance between gains and losses after breast cancer diagnosis
6	Construction of a coherent personal narrative
7	Development of new values and priorities of life
8	Redefinition of objectives and life goals

**Table 3:** Intervention program

Session 1 Psychoeducation and normalization of emotional reactions
Objectives: • Accept the negative reactions (e.g. fear, anxiety, anger, hopelessness, guilt, shame or confusion) as natural responses to the disease and understand the ambivalence between positive and negative feelings related to the personal experience of breast cancer. • Enhance the knowledge related to breast cancer, including, definition and disease progress, treatments, side effects and other procedures related to the disease. Activities: • “Emotions’ cards” – Each participant has to choose 6 cards from the total of cards with positive and negative emotions to illustrate which emotions that each participant felt during their own experience of breast cancer. This activity ends with a group discussion about the dichotomy between positive and negative emotions. • Psychoeducation – Psychologist provides information about breast cancer, including various topics. • Self-report measure – Each participant completes the questionnaire of basic beliefs [49].
Session 2 Facilitating emotional disclosure and communication
Objectives: • Increase emotional expression during breast cancer and practice communication skills for a well-adjusted expression of emotions and experiences related with breast cancer. Activities: • “Communication’ cards” – Each participant has to choose one set of cards that illustrate one hypothetic situation that address any communication issue. Each set of cards is composed by the situation, the positive and negative behavior, and the positive or negative outcome from the behavior. This activity ends with a group discussion about communication strategies and ways to promote an assertive style of communication. • Self-report measure - Each participant completes the Distress Disclosure Index (DDI) [57] and the Opener Scale [58, 59].
Session 3 Practice emotional self-regulation skills
Objectives: • Development of an adjusted stress management of individual emotions and reactions related to the disease, based on a more adaptive coping style. • Promote an autonomous use of self-regulation techniques. Activities: • Self-regulatory strategies for stress management – The psychologist introduces and promotes the practice of abdominal breathing and progressive muscle relaxation at the end of this session.
Session 4 Fears and concerns related to breast cancer
Objectives: • Improve the personal skills to an adjusted expression of concerns and expectations about the future, including, disease progression, treatment and even practical and financial concerns. Activities: • Rational Emotive Behavior Therapy [62] – Participants are invited to write their intrusive thoughts related with breast cancer and to write personal strategies to transform intrusive thoughts into deliberate thoughts. • Mindfulness – Psychologist introduces and promotes the practice of mindfulness exercises as a useful technique to change the intrusive thoughts.
Session 5 Balance between gains and losses after breast cancer diagnosis
Objectives: • Improve the balance between benefits and losses and the perception about the ambivalence between positive and negative feelings from the experience of breast cancer. Activities: • Balance of gains and losses – Each participant is invited to write their gains and losses in several areas of women's lives, as a result of their personal experience of breast cancer. A group discussion about the number of losses and gains reported by the group is promoted, at the end of the activity. • Challenge of core beliefs – To encourage the perception of possible changes in core beliefs, each participant is invited to wonder about core beliefs that she had before the diagnosis of breast cancer and that have been changed, as a result of the breast cancer experience.
Session 6 Construction of a coherent personal narrative
Objectives: • Construction of an individual narrative, to understand and integrate the experience of breast cancer in the set of the woman's life events [63]. • Promote the use of the expressive writing technique after the end of the intervention. Activities: • Expressive Writing - The group members received the following instructions: "Please indicate how breast cancer changed you and your personal life story". Promote group discussion about this topic. • Introduce expressive writing technique, its definition, objectives, and instructions. Each participant receives instructions for the expressive writing task along with all the materials required to perform this task at home. The written material is returned in the next session. The written information is confidential. All questions and doubts about this writing technique are clarified. The model used is according to Pennebaker [63], and the procedures are adapted from group interventions developed with cancer patients [60, 61].
Session 7 Development of new values and priorities of life
Objectives: • Expand the cognitive processing about core beliefs and personal values to achieve the redefinition of life priorities and the reevaluation of personal objectives, which are now consistent with the perceived identity changes. Activities: • “Reflection about principles of life” – Each participant is encouraged to recognize the previous principles of life and the new principles and objectives of life, more suited to the current reality, by answering the following question: "Please reflect on the principles of life that you have used through your life, until the present time."
Session 8 Redefinition of objectives and life goals
Objectives: • Redefinition of new life goals according to the actual personal narrative, which implies the rupture with the previous objectives, might occur, to give rise to life values more adjusted to the new reality and the current knowledge. Activities: • “Redefinition of life goals” – Participants are invited to write objectives, eventual obstacles and plan of action to achieve the objectives that they intend to achieve at medium or long-term. • “Problem-solving technique” – Psychologist introduces the problem-solving technique and promotes the development of problem-solving skills. • Self-report measure – Each participant completes the questionnaires from the second evaluation moment as well as a questionnaire to evaluate the intervention program.

### Data analysis

For statistical analysis, SPSS version 21 and AMOS version 21 software packages will be used. The descriptive analysis includes tabulating counts and frequencies of socio-demographic and clinical data, including breast cancer stage, cancer treatments, family cancer history, and personal history of disease. Bivariate analyzes will be used to assess the associations between socio-demographic factors and clinical information. To analyze the effects of treatment and to compare the control group with the intervention group, a Latent Growth Modeling will be used. To assess the relationship between the variables and to identify the predictors of primary outcomes, Multiple Regression Analysis and Structural Equation Modeling will be conducted. The level of significance will be set at α = .05.

## Discussion

Group interventions appear to be effective in promoting a better cognitive and emotional adjustment in diagnosed breast cancer patients. Furthermore, additional new group interventions have assessed PTG as an outcome [42–46]; however, thus far, this is the first study to investigate the effects of a group-based program with the explicit focus on promoting PTG in the psychosocial adjustment to breast cancer. This study will provide information on the efficacy of group-based interventions on PTG. The plan for the group sessions includes not only major cognitive processing about breast cancer, but also promotes the best opportunity to disclose emotions and share personal experiences, which facilitate the emergence of PTG.

The primary outcome of group interventions is to increase PTG at 6 and 12 months following the baseline assessment. We expect that potential significant differences among the three time points will occur in the intervention group. Further beneficial effects for cognitive processing, distress disclosure, PTSD, and social support are also evaluated, in addition to the potential mediation effect of psychosocial variables on PTG.

If PTG group intervention is found to be effective, it could be integrated into multidisciplinary daily clinical practice as a powerful way to promote PTG in women who are being treated for breast cancer. Furthermore, given that the intervention used cognitive-behavioral strategies, in addition to the positive effects on PTG, benefits may also be noted in the psychosocial adjustment to breast cancer, improvements in quality of life, greater adherence to therapy and to medical indications, and fewer hospital visits.

## Abbreviations

FCT: Portuguese national funding agency for science, research, and technology
PTG: posttraumatic growth
PTSD: posttraumatic stress disorder
RCT: randomized controlled trial
